# A genome-guided analysis of energy conservation in the thermophilic, cytochrome-free acetogenic bacterium *Thermoanaerobacter kivui*

**DOI:** 10.1186/1471-2164-15-1139

**Published:** 2014-12-18

**Authors:** Verena Hess, Anja Poehlein, Marie Charlotte Weghoff, Rolf Daniel, Volker Müller

**Affiliations:** Department of Molecular Microbiology & Bioenergetics, Institute of Molecular Biosciences, Johann Wolfgang Goethe University Frankfurt/Main, Max-von-Laue-Str. 9, 60438 Frankfurt, Germany; Department of Genomic and Applied Microbiology, Göttingen Genomics Laboratory, Georg August University, Institute for Microbiology and Genetics, Grisebachstraße 8, 37077 Göttingen, Germany

**Keywords:** Acetogen, *Thermoanaerobacter kivui*, Energy conservation, Genome sequence, H^+^ transport, Ech

## Abstract

**Background:**

Acetogenic bacteria are able to use CO_2_ as terminal electron acceptor of an anaerobic respiration, thereby producing acetate with electrons coming from H_2_. Due to this feature, acetogens came into focus as platforms to produce biocommodities from waste gases such as H_2_ + CO_2_ and/or CO. A prerequisite for metabolic engineering is a detailed understanding of the mechanisms of ATP synthesis and electron-transfer reactions to ensure redox homeostasis. Acetogenesis involves the reduction of CO_2_ to acetate *via* soluble enzymes and is coupled to energy conservation by a chemiosmotic mechanism. The membrane-bound module, acting as an ion pump, was of special interest for decades and recently, an Rnf complex was shown to couple electron flow from reduced ferredoxin to NAD^+^ with the export of Na^+^ in *Acetobacterium woodii*. However, not all acetogens have *rnf* genes in their genome. In order to gain further insights into energy conservation of non-Rnf-containing, thermophilic acetogens, we sequenced the genome of *Thermoanaerobacter kivui*.

**Results:**

The genome of *Thermoanaerobacter kivui* comprises 2.9 Mbp with a G + C content of 35% and 2,378 protein encoding orfs. Neither autotrophic growth nor acetate formation from H_2_ + CO_2_ was dependent on Na^+^ and acetate formation was inhibited by a protonophore, indicating that H^+^ is used as coupling ion for primary bioenergetics. This is consistent with the finding that the *c* subunit of the F_1_F_O_ ATP synthase does not have the conserved Na^+^ binding motif. A search for potential H^+^-translocating, membrane-bound protein complexes revealed genes potentially encoding two different proton-reducing, energy-conserving hydrogenases (Ech).

**Conclusions:**

The thermophilic acetogen *T. kivui* does not use Na^+^ but H^+^ for chemiosmotic ATP synthesis. It does not contain cytochromes and the electrochemical proton gradient is most likely established by an energy-conserving hydrogenase (Ech). Its thermophilic nature and the efficient conversion of H_2_ + CO_2_ make *T.* *kivui* an interesting acetogen to be used for the production of biocommodities in industrial micobiology. Furthermore, our experimental data as well as the increasing number of sequenced genomes of acetogenic bacteria supported the new classification of acetogens into two groups: Rnf- and Ech-containing acetogens.

## Background

Acetogenic bacteria represent an ecologically important group of anaerobes that are ubiquitous in nature [1]. They constitute the penultimate limb in the anaerobic food web and convert a number of substrates exclusively to acetate, which is then converted by methanogenic archaea to methane, the final product of anaerobic food webs [2]. Acetogens convert hexoses to three moles of acetate according to:
1

indicating the presence of a pathway that is able to reduce two moles of CO_2_ to acetate [3]. This pathway has been elucidated mainly in the thermophilic species *Moorella thermoacetica* (formerly *Clostridum thermoaceticum*) and is named according to its discoverers as the Wood-Ljungdahl pathway (WLP) [4]. It involves two branches and each of them contributes to the reduction of one molecule of CO_2_. In the carbonyl branch, CO_2_ is reduced to enzyme-bound CO by the CO dehydrogenase/acetyl-CoA synthase (CODH/ACS). Electrons for this reduction are provided by reduced ferredoxin [5–7]. In the methyl branch of the WLP, another molecule CO_2_ is reduced first to formate, which is then activated to formyl-THF in an ATP-dependent reaction [8, 9]. Subsequently, formyl-THF is converted to methenyl-THF and then reduced stepwise to methyl-THF *via* methylene-THF. Eventually, the methyl group and the enzyme-bound CO are joined to form acetyl-CoA, catalyzed by the key enzyme of the WLP, the CODH/ACS [5–7, 10–12]. Subsequently, acetyl-CoA is converted to acetyl-phosphate and acetate [13, 14]. The latter step involves the production of ATP by an acetate kinase. However, since one mole of ATP is consumed during activation of formate, there is no net ATP gain by substrate level phosphorylation in the WLP. The WLP also enables lithotrophic growth of acetogens on H_2_ + CO_2_ according to:
2

and, therefore, additional energy must be conserved by a chemiosmotic mechanism. The chemiosmotic process of energy conservation was recently uncovered in *Acetobacterium woodii*. A soluble, electron bifurcating, ferredoxin- and NAD^+^- reducing hydrogenase oxidizes molecular hydrogen and generates a reduced electron carrier with a very low redox potential, ferredoxin (E^0^’ ≈ −500 mV; a redox potential in this range has to be assumed since a ferredoxin is the electron donor for the CO_2_/CO couple E^0^’ = −520 mV). Reduced ferredoxin is then oxidized by a membrane-bound ferredoxin:NAD^+^-oxidoreductase encoded by the *rnf* genes and therefore also termed Rnf complex. The Rnf complex is composed of six subunits that harbor (covalently-bound) flavins and iron sulfur centers as electron carriers, and the electrons are transferred to the acceptor NAD^+^ [15]. Electron transfer from reduced ferredoxin to NAD^+^ is exergonic and this electron transfer is used to expel sodium ions from the cytoplasm thus generating an electrochemical sodium ion gradient across the membrane [16]. The electrochemical Na^+^ gradient then drives the synthesis of ATP *via* a Na^+^-F_1_F_O_ ATP synthase [17, 18]. The energy-conserving module in *A. woodii* thus comprises only one coupling site, the Rnf complex, and a Na^+^-F_1_F_O_ ATP synthase. Acetogenesis in *A. woodii* therefore has a modular appearance: An energy-conserving module that is connected to the WLP module by soluble electron carriers such as pyridine nucleotides and ferredoxin. The WLP module is not energy conserving, it serves the function to reoxidize the end products of the anaerobic respiration and to provide acetyl-CoA for biomass synthesis [19].

The use of two different modules for energy conservation and re-oxidation of reduced electron carriers (WLP) opens interesting possibilities for biotechnological applications. In principal, any reductive pathway can be coupled to the energy-conserving module and indeed, acetogens are known that reduce nitrate [20, 21], phenylacrylates [22, 23] and fumarate [24, 25], or acetyl-CoA to ethanol [26] or butyrate [27, 28]. Very recently, the genetic coupling of a butanol-production pathway to the energy-conserving module proved successful in *Clostridium ljungdahlii* [29]. Production of biocommodities at high temperatures has several advantages such as lower costs for cooling and distillation or lower risk of contaminations. The genome of the thermophilic acetogen *M. thermoacetica* (T_opt_: 55-60°C) has been sequenced [30] and the way(s) of energy conservation can be predicted from the genome as well as experimental analyses [31]. Unfortunately, this thermophilic model strain grows poorly on H_2_ + CO_2_ (doubling times of up to 24 h [32]), a substrate used for 3rd generation biotechnology, thus limiting its use in industrial microbiology. In contrast, the acetogen *Thermoanaerobacter kivui* grows very fast on H_2_ + CO_2_ with doubling times around 2 h and has an even higher T_opt_ of 66°C [33]. Moreover, its acetate to biomass ratio was about half compared to *M. thermoacetica* indicating a more efficient way to conserve energy [34]. This is also exemplified by yield measurements: 97% more biomass is produced from one mole of H_2_ in *T. kivui* compared to *M. thermoacetica* [34]. This prompted us to sequence the genome of *T. kivui* to predict by bioinformatic analyses followed by experimental analyses how this acetogen couples acetogenesis to chemiosmotic energy conservation.

## Methods

### Growth conditions

*T. kivui* LKT-1 (DSM 2030) was grown at 65°C in medium that was prepared as described by Leigh *et al*. [33] with some modifications. Complex medium contained: 50 mM NaH_2_PO_4_, 50 mM Na_2_HPO_4_, 1.2 mM K_2_HPO_4_, 1.6 mM KH_2_PO_4_, 4.7 mM NH_4_Cl, 1.7 mM (NH_4_)_2_SO_4_, 7.5 mM NaCl, 0.37 mM MgSO_4_, 42 μM CaCl_2_, 7.2 μM Fe(II)SO_4_, 54 mM NaHCO_3_, 3 mM cysteine-HCl, 0.2% [w/v] yeast extract, 1.0% [v/v] trace element solution (DSM 141), 1.0% [v/v] vitamin solution (DSM 141), and 4 μM resazurin. Glucose was added as a carbon source from an autoclaved anoxic stock solution to a final concentration of 28 mM. All vessels were pressurized with 1 bar N_2_:CO_2_ (80:20 [v/v]). Growth experiments for determination of Na^+^-dependence of autotrophic growth were performed exactly as described by Yang & Drake [35]. The contaminating amount of Na^+^ was 160 ± 4 μM, determined using an Orion Star A214 Na^+^-selective electrode (Thermo Scientific, USA). Growth was determined by measuring the optical density at 660 nm (OD_660_) with a spectrophotometer.

### Preparation of membranes from *T. kivui*

Cells were grown to an optical density at 600 nm of 1.9 – 2.4 as described in 2 × 500 ml complex medium in 1-l-flasks (Glasgerätebau Ochs, Bovenden-Lenglern, Germany) with 28 mM glucose as carbon source and harvested anaerobically by centrifugation at 11,500 × g for 10 min at 4°C. Cells were washed once in buffer A (50 mM Tris, 20 mM MgSO_4_, 20% glycerol, 2 mM DTE, 4 μM resazurin, pH 7.5) and, after another centrifugation step, resuspended in 5–10 ml buffer A. Cells were disrupted by a single passage through a French press (110 MPa). Cell debris and whole cells were removed by a centrifugation step for 30 minutes at 23,700 × g and 4°C. The cell extract was separated into the cytoplasmic and membrane fraction by ultracentrifugation (150,000 × g, 2 h, 4°C). The membranes were washed once in buffer A and sedimented again *via* ultracentrifugation. Finally, the membranes were resuspended in ~ 5 ml buffer A and used immediately for the measurement of Fd_red_:NAD^+^ oxidoreductase activity. Protein concentrations were determined as described previously [36].

### Measurement of Fd_red_:NAD^+^ oxidoreductase activity

Measurement of electron transfer from reduced ferredoxin to NAD^+^ was performed as described [37] at 60°C in anaerobic cuvettes filled with 1 ml 20 mM Tris–HCl buffer (pH 7.7) containing 20 mM NaCl, 2 mM DTE and 4 μM resazurin at a pressure of 0.5 × 10^5^ Pa CO. Ferredoxin (30 μM; purified from *C. pasteurianum* as described [38]), CODH/ACS (30 μg/ml; purified from *A. woodii* as described [37], and washed membranes (150 μg/ml) were added. The reaction was started by addition of NAD^+^ (4 mM). Formation of NADH was measured at 340 nm.

### Isolation of chromosomal DNA

Chromosomal DNA of *T. kivui* was isolated according to the procedure described [39] and modified by Ausubel *et al*. [40].

### Sequencing strategy

The genome of *T. kivui* was sequenced with a combined approach using the 454 GS-FLX Titanium XL system (Titanium GS70 chemistry, Roche Life Science, Mannheim, Germany) and the MiSeq (Illumina, San Diego, CA). Shotgun libraries were prepared according to the manufacturer’s protocols, resulting in 110,391 reads for 454 shotgun sequencing and 5,916,460 150-bp paired-end reads for Illumina sequencing. All of the 454 shotgun reads and 1 Mio 150-bp paired-end Illumina reads were used for the initial hybrid *de novo* assembly with MIRA 3.4 [41] and Newbler 2.8 (Roche Life Science, Mannheim, Germany). The final assembly contained 42 contigs with an average coverage of 89.20. For scaffolding and contig ordering tasks we used the Move Contigs tool of the Mauve Genome Alignment Software [42] and the genomes of *T. mathranii mathranii* A3, DSM 11426, *T. brockii finii* Ako-1, DSM 3389 and *T. wiegelii* Rt8.B1 as references. Additionally, contigs that could not be ordered with Mauve were examined *via* Gene Ortholog Neighborhoods based on bidirectional best hits implemented at the IMG-ER (Integrated Microbial Genomes-Expert Review) system [43, 44] and with multiplex PCR [45]. Sequence gaps were closed in the Gap4 (v.4.11) software of the Staden Package [46] by PCR-based techniques and primer walking with conventional Sanger sequencing, using BigDye 3.0 chemistry on an ABI3730XL capillary sequencer (Applied Biosystems, Life Technologies GmbH, Darmstadt, Germany).

### Gene prediction and annotation and analysis

The software tool prodigal (**P**rokaryotic **D**ynamic **P**rogramming **G**enefinding **A**lgorithm) [47] was used for automatic gene prediction, while identification of rRNA and tRNA genes was performed with RNAmmer and tRNAscan, respectively [48, 49]. Automatic annotation was carried out with the IMG-ER (Integrated Microbial Genomes-Expert Review) system [43, 44], but annotation was afterwards manually curated by employing BLASTP and the Swiss-Prot, TrEMBL, and InterPro databases [50]. Prophage regions were identified using the online tool PHAST [51].

### Experiments with resting cells and acetate determination

*T. kivui* was grown as described in 2 × 500 ml complex medium with 28 mM glucose as carbon source to an OD_600_ of 1.9 to 2.4. The culture was centrifuged anaerobically at 11,500 × g and 4°C for 10 min. Cells were washed twice in imidazole buffer (50 mM imidazole, 20 mM MgSO_4_, 20 mM KCl, 4 mM DTE, 4 μM resazurin, pH 7.0). After the last centrifugation step, cells were resuspended in 2 ml imidazole buffer. The protein concentration was determined according to [52], being approx. 100 mg/ml. In order to determine the conversion of H_2_ + CO_2_ to acetate, 100-ml-serum flasks (Glasgerätebau Ochs GmbH, Bovenden-Lenglern, Germany) were filled with 10 ml imidazole buffer containing 50 mM KHCO_3_. If indicated, NaCl was added to a concentration of 20 mM. If applied, the ionophores ETH2120 and TCS were added to a concentration of 30 μM each. Subsequently, the gas phase of the serum flasks was exchanged to 1 bar H_2_ + CO_2_ (80:20 [v/v]) and then preheated to a temperature of 65°C in a water bath. The reaction was started by addition of resting cells to a final concentration of 1 mg/ml. Henceforth, 500-μl-samples were taken at time points as indicated. Samples were centrifuged immediately at 18,000 × g for 1 min and the supernatant was stored at −20°C. Determination of the acetate concentration of all samples was carried out using a commercially available kit (Acetic acid, Co. R-Biopharm, Darmstadt, Germany).

### Nucleotide sequences and accession number

The sequence data described here have been deposited in GenBank under Acession No. CP009170 [GenBank:CP009170].

## Results

### General features of the *T. kivui*genome

The complete genome of *T. kivui* (accession number CP009170) consists of a circular chromosome with a size of 2.397 Mbp and an overall G + C content of 35.06 mol%. General features of the genome are listed in Table 1. We could identify 2378 putative protein-coding genes, three complete rRNA clusters and 58 tRNA genes. The tRNA necessary for incorporation of selenocystein is also present, but this organism is probably not able to assemble selenoproteins, since the *selABC* gene cluster coding for essential proteins could not be identified, only a very short fragment of *selB* is present. 76.05% (1810) of the open reading frames (ORF) could be assigned to a putative function, 568 ORFs (23.86%) were annotated as hypothetical proteins and 35 ORFs as pseudo genes. Approximately 61% (1551 ORFs) of all protein-encoding genes could be assigned to at least one of the 21 functional COGs (Cluster of Orthologous Groups). The two most abundant categories were “general function” and “amino acid transport and metabolism”, to which 10.24% and 9.18%, respectively, could be assigned to, followed by “function unknown”, “translation, ribosomal structure and biogenesis”, “coenzyme transport and metabolism“, “replication, recombination and repair” and “energy production and conversion” with 9.06%, 8.30%, 6.83%, 6.65% and 6.00%, respectively.

**Table 1:** **General features of the** ***T. kivui*** **genome**

	*T. kivui*
Genome size	2397289 bp
Protein encoding orfs	2378
Pseudo genes	35
Percent coding (%)	87.55
G + C content (mol%)	35.06
rRNA	10
tRNA	58
CDS with function prediction	2024
CDS without assigned function	356
CDS assigned to COGs	1559
CRISPR count	3

As mentioned before, “replication, recombination and repair” is one of the most abundant COG category in the genome of *T. kivui*. Proteins which were assigned to this category are Cas proteins (CRISPR-associated sequences) and the encoding genes are often located adjacent to CRISPR loci (Clustered Regularly Interspaced Short Palindromic Repeats). The CRISPR/Cas system protects the genome against invading mobile elements such as plasmids or phages and consists of contiguous repeats with different length (20 to 47) and different numbers [53–55]. The genome of *T. kivui* harbors 3 CRISPR loci with 35, 48 and 21 repeats, respectively. The first CRISPR locus is flanked by four genes which could be assigned to a III-B/polymerase-RAMP module CRISPR/Cas system subtype (TKV_c14620-TKV_c14650), the second locus is upstream flanked by a type III-B/polymerase-RAMP module subtype (TKV_c23500-TKV_c23560) and downstream by a hybrid cluster consisting of I-B/Teanap-Hmari and again a III-B/polymerase-RAMP module subtype where the *cmr2* gene is interrupted by a transposase (TKV_c23590-TKV_c23770), while a second I-B/Teanap-Hmari subtype CRISPR/Cas system is located adjacent to the third CRISPR locus (TKV_c24261). In addition we could identify a complete subtype III-A CRISPR/Cas system (TKV-c17330-TKV_c17390), which is not associated with a CRISPR locus. All CRISPR/Cas clusters were annotated according to the polythetic classification of CRISPR/Cas systems [56]. There is no complete prophage located on the chromosome of *T. kivui*.

### Substrate utilization

*T. kivui* can not only grow autotrophically on H_2_ + CO_2_ (or formate) but also heterotrophically on glucose, fructose, mannose, and pyruvate [33]. Correspondingly, genes coding for all enzymes for glycolysis were found in the *T. kivui* genome with only the hexokinase and the aldolase being encoded twice (Table 2). Despite the gene cluster TKV_c16300-TKV_c16340 (coding for the conversion of glyceraldehyde-3-P to phosphoenolpyruvate), the genes are spread over the genome. Fructose is taken up and phosphorylated to fructose-1-P by a fructose-specific PTS system (TKV_c23130 and TKV_c23140). Interestingly, the genes encoding component IIB and IIC are fused to one gene, TKV_c23130. Subsequently, fructose-1-P is phosphorylatedtofructose-1,6-bisphosphatebyFruK (TKV_c23150), a 1-phosphofructokinase. On the other hand, mannose is taken up by a second, mannose-specific PTS system (TKV_c06180-TKV_c06200) and thereby activated to mannose-6-P. This substrate is converted to fructose-6-P by a mannose-6-P isomerase (TKV_c16180). Glycolysis results in the formation of pyruvate, which is most probably converted to acetyl-CoA by use of the pyruvate:ferredoxin oxidoreductase. A gene cluster (TKV_c19290-TKV_c19260) encoding this enzyme was found in the genome of *T. kivui*, which encodes a ferredoxin, as well as the α, β, and γ subunit of the complex. Eventually, the phosphotransacetylase (TKV_c13970) and acetate kinase (TKV_c13960) catalyze the conversion of acetyl-CoA to acetate. Although two potential alcohol deyhdrogenases (TKV_c02600 and TKV_c22590) are encoded in the genome, an aldehyde dehydrogenase was not found. This is in accordance with the finding that ethanol was not detected in cells grown on H_2_ + CO_2_, glucose or pyruvate [33].

**Table 2:** **Genes encoding enzymes of the Embden-Meyerhof-Parnas pathway**

Enyzme	Locus tag
Hexokinase	TKV_c00920, TKV_c17910
Glucose-6-P isomerase	TKV_c16780
6-phosphofructokinase	TKV_c16900
Aldolase	TKV_c01430, TKV_c04080
Triose-P isomerase	TKV_c16320
Glycerinaldehyde-3-P deyhdrogenase	TKV_c16340
Phosphoglycerate kinase	TKV_c16330
Phosphoglycerate mutase	TKV_c16310
Enolase	TKV_c16300
Pyruvate kinase	TKV_c16890

### Genes involved in the Wood-Ljungdahl pathway

**Figure 1:**
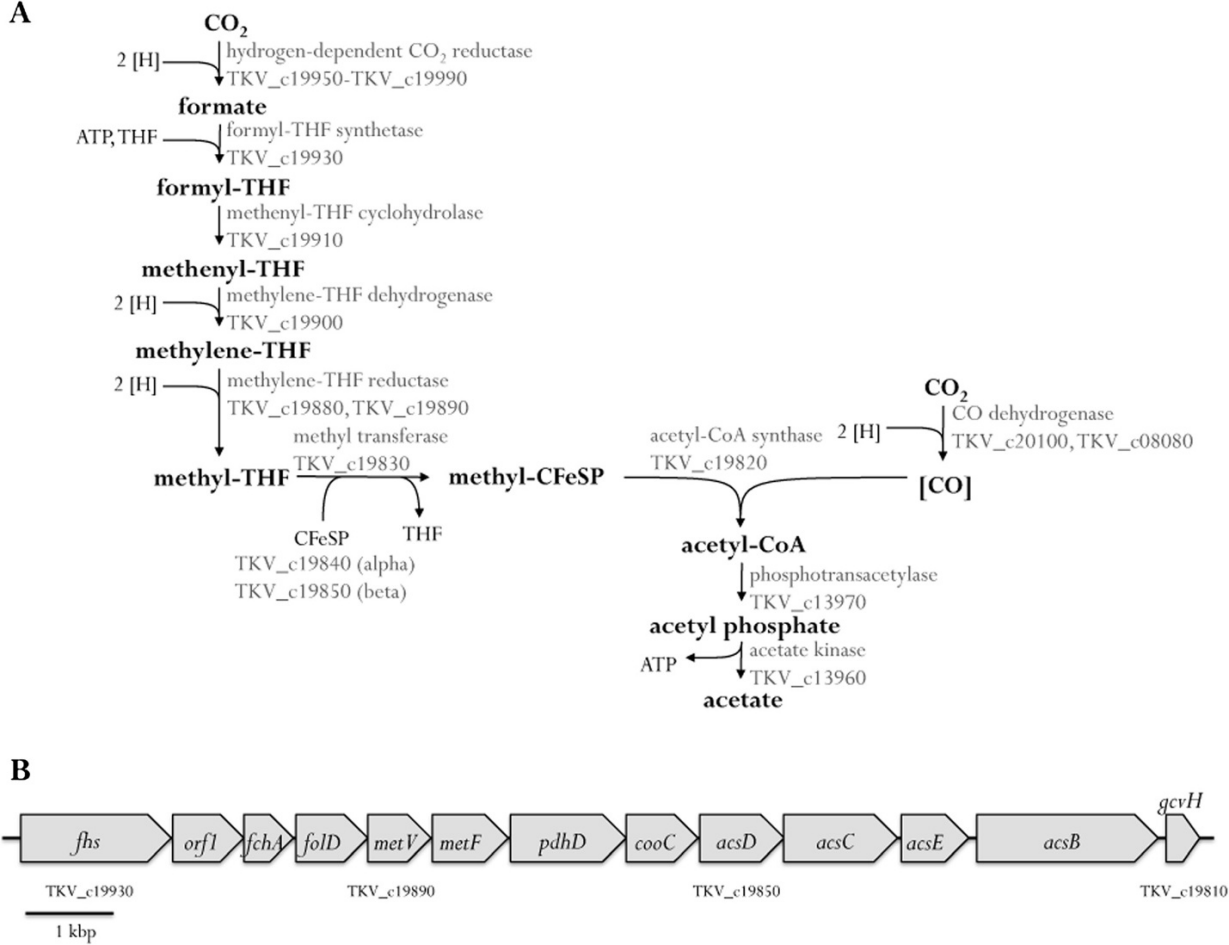
**The Wood-Ljungdahl pathway in** ***T. kivui*** **.** The encoding genes are indicated **(A)** and the genetic organization is shown in **(B)**. CoFeSP, corrinoid/FeS protein; THF, tetrahydrofolate; [H], reducing equivalent, corresponding to one electron; *orf1*, probably encoding a thymidylate synthase.

The genes encoding the key enzymes in the Wood-Ljungdahl pathway of *T. kivui* are shown in Figure 1A. The first step in the methyl branch of the WLP is the reduction of CO_2_ to formate by a formate dehydrogenase. A corresponding gene cluster very similar to the one of *A. woodii* was found on the chromosome of *T. kivui* (TKV_c19950-TKV_c19990). In *A. woodii*, the cluster consists of 7 genes [57], with *fdhF2* being an isogene of *fdhF1*, encoding a selenium-containing Fdh. A gene encoding an electron transfer protein (*hycB*) follows each *fdh* gene. In *T. kivui*, only a selenium-free Fdh (*fdhF*/TKV_c19990) is encoded in the cluster. This gene is followed by *hycB3* (TKV_c19980) and *hycB4* (TKV_c19970), both encoding small FeS proteins. Adjacent to *hycB4* is a gene (*hydA2*/TKV_c19960) encoding a hydrogenase subunit. Both clusters also encode a gene annotated as *fdhD*. Since the enzyme purified from *A. woodii* did not contain an FdhD subunit [58], its function remains to be elucidated. In *A. woodii*, this enzyme complex was recently shown to catalyze the hitherto unknown reduction of CO_2_ with electrons coming directly from molecular hydrogen and was therefore named hydrogen-dependent CO_2_ reductase (HDCR) [58]. Thus, it is likely that *T. kivui* reduces CO_2_ to formate with H_2_ as well. This is in contrast to the close phylogenetic neighbor *M. thermoacetica*, which was shown to reduce CO_2_ with electrons coming from NADPH [59]. Many of the genes encoding functions of the WLP are located in one main gene cluster (Figure 1B), which lies in close proximity to the genes coding for the hydrogen-dependent CO_2_ reductase. The product of the first gene of the operon, *fhs* (TKV_c19930), probably catalyzes the initial ATP-dependent activation of formate to formyl-THF. Subsequent to *fhs* is an open reading frame (*orf1*), whose product is similar to thymidylate synthases. Adjacent to *orf1* are genes encoding the methenyl-THF cyclohydrolase (*fchA*), methylene-THF dehydrogenase (*folD*) as well as two subunits of the methylene-THF reductase (*metV* and *metF*). Those are followed by a dihydrolipoamide dehydrogenase (*pdhD*), a maturation factor of the CO dehydrogenase (*cooC*), subunit beta (*acsD*) and subunit alpha (*acsC*) of the corrinoid iron-sulfur protein. The putative operon is completed by genes encoding a methyltransferase (*acsE*), an acetyl-CoA synthase (*acsB*) and protein H of a glycine cleavage system (*gcvH*). A possible function of GcvH within the WLP is unclear, but since this gene is also found within this gene cluster in *A. woodii* [57] and *M. thermoacetica* [30], one cannot exclude a so far undiscovered function of this enzyme within the pathway. Thus, as in *A. woodii*, proteins for the conversion of formate to methyl-THF are encoded by one gene cluster. However, in *A. woodii folD* and *metV* do not lie adjacent to each other but are separated by the gene *rnfC2*, probably coding for a third subunit of the methylene-THF reductase in this organism [57]. In *M. thermoacetica*, the genes coding for proteins of the methyl branch of the WLP are spread all over the genome. Only recently, MetF and MetV were shown to form a complex with the proteins HdrABC and MvhD [31]. Such genes were not found in *T. kivui*, implicating that the soluble methylene-THF reductase is composed of only 2 subunits. In *A. woodii* and *M. thermoacetica*, a separate gene cluster coding for the methyltransferase AcsE as well as subunits of the CODH/ACS is located somewhere else on the genome. In *T. kivui*, only two genes encoding a CO dehydrogenase (*acsA*/TKV_c20100 and *cooS/*TKV_c08080), a phosphotransacetylase and an acetate kinase are encoded elsewhere on the genome. Thus the WLP gene cluster is outstanding, since it encodes all proteins (despite AcsA, which is separated by 10 genes) that are required for the conversion of CO_2_ to acetyl-CoA.

### Transfer of reducing equivalents by soluble enzymes

In all acetogens examined, ferredoxin and NAD(P) serve as common electron carriers in the WLP and in energy conservation. Reducing equivalents required in the WLP of *T. kivui* may be delivered from molecular hydrogen by a soluble electron bifurcating hydrogenase HydABC complex (TKV_c19580-TKV_c19600) similar to the electron bifurcating hydrogenases of *A. woodii* and *M. thermoacetica*, which reduce NAD^+^ and ferredoxin in equimolar amounts [60–62]. HydA1 (TKV_c19850) of *T. kivui* shows 56% and 49% identity to the corresponding subunits of *M. thermoacetica* and *A. woodii*, respectively. The identities for HydB (TKV_c19590) to the homologous proteins of *M. thermoacetica* and *A. woodii* are 54% and 59%, the ones for HydC (TKV_c19600) amount 42% and 47%, respectively. Apart from HydA2 of the HDCR and the HydABC complex, there is no other soluble hydrogenase encoded in the genome of *T. kivui*.

As also found in *M. thermoacetica* [32], the genome of *T. kivui* encodes an electron bifurcating transhydrogenase (NfnAB complex), which is encoded by the genes TKV_c22270 and TKV_c22280. TKV_ c22270 shows 57% and TKV_ c22280 69% identity to NfnA (Moth_1518) and NfnB (Moth_1517), respectively, from *M. thermoacetica.* In *M. thermoacetica*, this enzyme complex coupled the endergonic transfer of electrons from NADH to NADP^+^ to the exergonic reduction of NADP^+^ with Fd_red_ [32].

### F_1_F_O_ ATP synthase of *T. kivui*

ATP synthases consist of a soluble head domain, that catalyzes ATP synthesis or hydrolysis and a membrane domain that is responsible for ion translocation [63]. Genes encoding all the subunits of a F_1_F_O_ ATP synthase were found in the genome of *T. kivui* in one gene cluster (TKV_c06410-TKV_c06480). Interestingly, the cluster does not code for the gene *atpI*, which is the first gene of most bacterial *atp* operons and was shown to be essential for the assembly of the *c* ring in *A. woodii* [64]. The genes flanking the ATP synthase gene cluster of *T. kivui* apparently do not code for proteins that are required for ATP synthesis.

**Figure 2:**
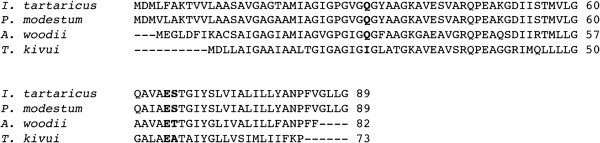
**Sequence alignment of** ***c*** **subunits of Na** ^**+**^ **-dependent ATP synthases and the subunit** ***c*** **(TKV_c06420) of** ***T. kivui*** **.** The Na^+^ binding motif is highlighted in bold.

The membrane-integral *c* subunit of F_1_F_O_ ATP synthases determines the ion specificity of the enzyme. To check whether the *c* subunit of *T. kivui* has a conserved Na^+^ binding site, sequence alignments of several *c* subunits of Na^+^-dependent ATP synthases along with the one of *T. kivui* (*atpE*/TKV_c06420) were performed. As can be seen in Figure 2, the Na^+^-dependent F_1_F_O_ ATP synthases from *Ilyobacter tartaricus*, *Propionigenium modestum* and *A. woodii* have the conserved Na^+^ binding motif consisting of the amino acids Q…..ES/T [65, 66]. This motif cannot be found in the *c* subunit of *T. kivui* (Figure 2). Here, the glutamine (Q) is changed to an isoleucine (I) and instead of the serine (S) or threonine (T), there is an alanine (A). Since the proton-binding site (E55) of the *c* subunit is well conserved in *T. kivui*, this finding suggests that the F_1_F_O_ ATP synthase of *T. kivui* is H^+^-dependent. This finding is in line with the hypothesis that the bioenergetics of *T. kivui* is not based on a sodium ion but proton current across its cytoplasmic membrane. To substantiate this hypothesis, the effect of Na^+^ on growth and acetate formation was analyzed.

### Autotrophic growth is not Na^+^-dependent

**Figure 3:**
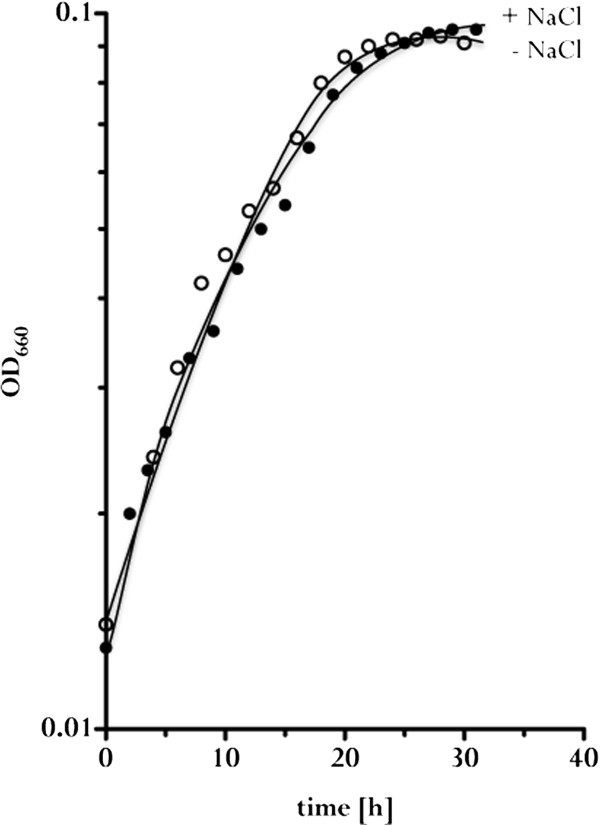
**Effect of Na** ^**+**^ **on autotrophic growth of** ***T. kivui*** **.** Cultures grown on H_2_ + CO_2_ were transferred into Na^+^-enriched (●) and Na^+^-deficient (○) minimal medium. The curves shown are representative for three independent experiments. Precultures were grown for four transfers in the same medium.

In order to determine a Na^+^ dependence of *T. kivui* during autotrophic growth, cells were transferred four times in Na^+^-depleted minimal medium, the contaminating amount of Na^+^ was determined to 160 ± 4 μM, Na^+^-enriched medium contained 71 ± 1 mM NaCl. As evident from Figure 3, the growth rate and the final optical density when growing on H_2_ + CO_2_ were independent of the Na^+^ concentration. Both cultures grew to an OD_600_ of ~0.09 within 30 hours. These data strongly support our hypothesis that *T. kivui* is not Na^+^- but H^+^-dependent.

### Acetate formation from H_2_ + CO_2_ is H^+^-dependent

**Figure 4:**
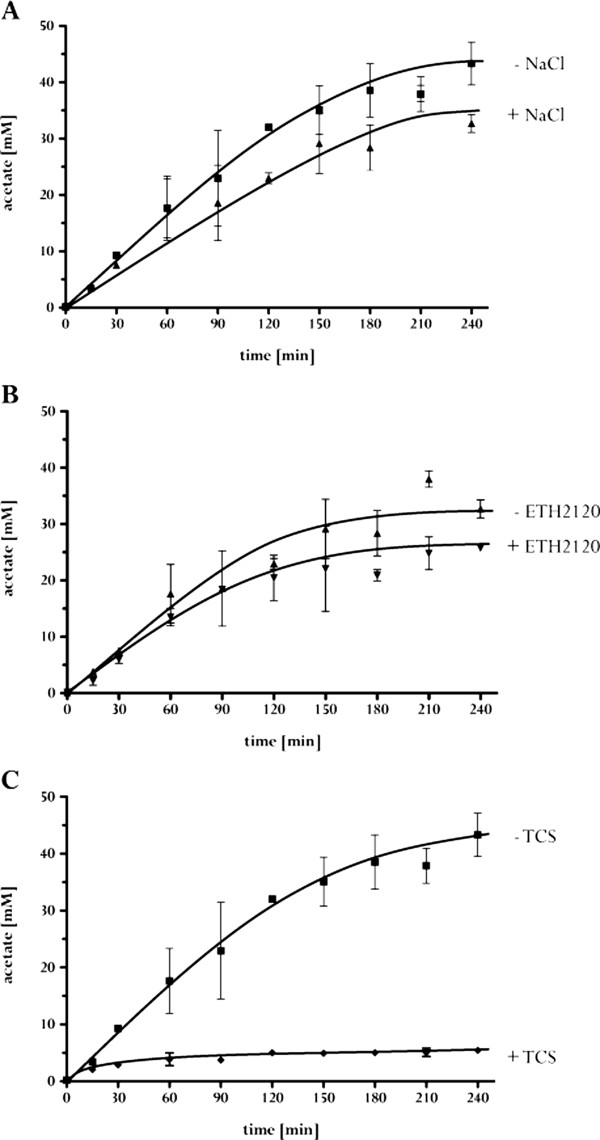
**Acetate formation from H**_**2**_ **+ CO**_**2**_ **by resting cells of** ***T. kivui*** **is inhibited by TCS.** Whole cells of *T. kivui* were incubated with H_2_ + CO_2_ in buffer containing 50 mM imidazole, 20 mM MgSO_4_, 20 mM KCl, 50 mM KHCO_3_ and 4 mM DTE (pH 7.0). **A**: 350 ± 20 μM (■) or 20 mM (▲) NaCl; **B**: 20 mM NaCl with (▼) or without (▲) 30 μM ETH2120; **C**: 350 ± 20 μM NaCl with (♦) or without (■) 30 μM TCS. The final protein concentration of the resting cells in the assay was 1 mg/ml. All values are mean from three replicates.

In order to identify the nature of the coupling ion in acetogenesis of *T. kivui* irrevocably, cell suspensions of glucose-grown cells were prepared and acetate formation from H_2_ + CO_2_ was determined. As evident from Figure 4A, the presence of NaCl had almost no effect on the final amount and rate of acetate formation, arguing against Na^+^ as coupling ion. In contrast, the addition of NaCl even led to a slightly decreased acetate production rate. Without NaCl present in the system, the final amount of acetate was about 42 mM, whereas only 35 mM acetate were produced in the presence of 20 mM NaCl. The contaminating amount of Na^+^ was 350 ± 20 μM.

Although the experimentally determined Na^+^ concentration in the assay without added NaCl is well below the *K*_M_ for Na^+^ during acetate formation in *A. woodii*, it may well be that the *K*_M_ for Na^+^ in *T. kivui* is lower than the contaminating Na^+^ concentration in the assay. To determine whether acetogenesis in *T. kivui* relies on a transmembrane Na^+^ or H^+^ gradient, the effect of different ionophores on acetate formation was monitored. The Na^+^ ionophore ETH2120 had almost no effect on acetate formation (in the absence or presence of sodium ions) (Figure 4B), whereas the protonophore TCS led to a complete inhibition of acetate formation. Here, the final amount of acetate was only 5 mM (Figure 4C). The experiments with resting cells of *T. kivui* give strong evidence that *T. kivui* does not belong to the Na^+^-dependent acetogens, but that H^+^ is the coupling ion.

### Membrane-embedded electron transfer coupled to the formation of a transmembrane proton gradient

**Figure 5:**
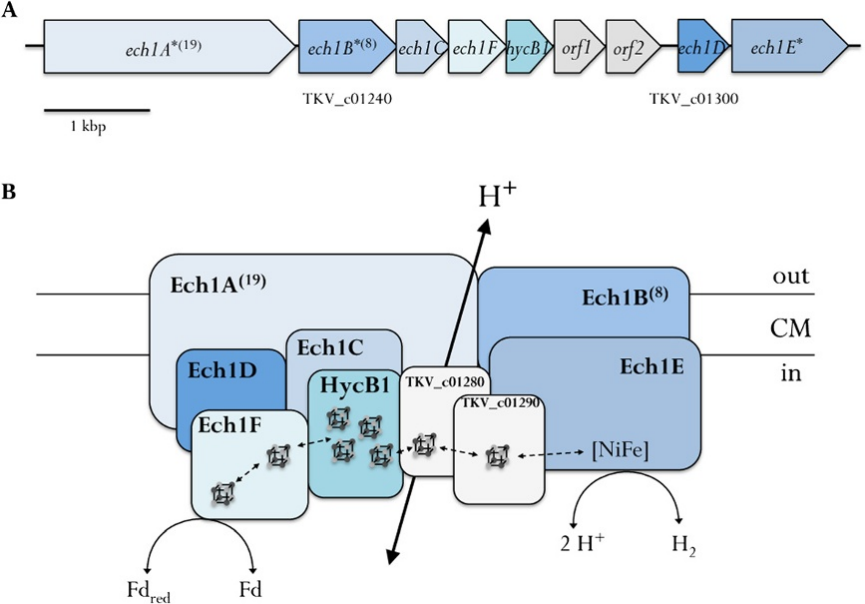
**Arrangement of genes in the cluster (A) and model (B) of the Ech-type complex Ech1.** Electron flow from reduced ferredoxin to H^+^ and the coupled export of protons is shown. FeS clusters are indicated. *, predicted transmembrane protein (number of transmembrane helices); Fd, ferredoxin; *orf1*/*orf2*, encoding small FeS containing proteins (TKV_c01280 and TKV_c01290) with similarity to the N-terminus of MetV.

**Figure 6:**
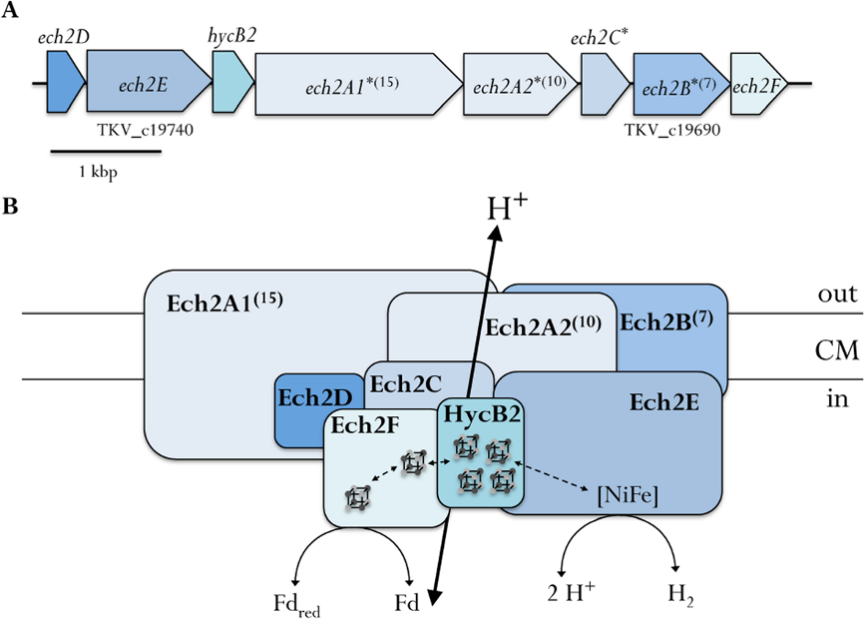
**Arrangement of genes in the gene cluster (A) encoding a second potential Ech-type complex Ech2 (B).** FeS clusters are indicated. *, predicted transmembrane protein (number of transmembrane helices); Fd, ferredoxin.

In *A. woodii*, the Na^+^-translocating Rnf complex is the only coupling site [15, 16, 37]. However, inspection of the genome and the absence of Fd_red_:NAD^+^ oxidoreductase activity at membranes of *T. kivui* (data not shown) revealed that *T. kivui* does neither possess an Rnf complex nor a complex with similar function. Furthermore, no genes encoding for cytochrome synthesis could be detected in the genome. Therefore, the genome of *T. kivui* was sought for genes encoding potential ion pumping membrane-bound oxidoreductases. As in *M. thermoacetica*, two gene clusters with similarities to Ech-type complexes were found: TKV_c01230-TKV_c01310 (Figure 5A) and TKV_c19750-TKV_c19680 (Figure 6A). Ech complexes are thought to be the ancestor of complex I [67] and although final proof with a purified enzyme is still pending, strong indications were given that Ech complexes in methanogens couple the electron transfer from reduced ferredoxin to H^+^ with the translocation of H^+^ across the cytoplasmic membrane [68, 69]. However, in *M. thermoacetica*, one Ech-type complex might be coupled to a formate dehydrogenase (Moth_2183) and the second does not possess all residues essential for a catalytic [NiFe] domain in the large hydrogenase subunit EchE (Moth_0980).

The first gene of the first *ech* gene cluster of *T. kivui*, *ech1A* (TKV_c01230), codes for a transmembrane protein with 19 predicted transmembrane helices with a weak similarity (17%) to EchA from *Methanosarcina barkeri*. The identity to homologous proteins of *M. thermoacetica* is 21% to Moth_2191, 23% to Moth_2188 and Moth_2187 (encoded by the first *ech* cluster), as well as 20% to Moth_0985 and 21% to Moth_0986 and Moth_0987 (encoded by the second *ech* cluster). The product of the following gene, *ech1B*, has 8 transmembrane helices and shows 21% identity to *M. barkeri* EchB, the identity to homologous proteins from *M. thermoacetica* is 22% for Moth_2190 and 26% for Moth_0981. Both subunits are thought to be involved in H^+^ transport [70, 71]. *ech1C* codes for the small hydrogenase subunit, probably involved in electron transfer. The identity to *M. barkeri* EchC is 38%, to *M. thermoacetica* Moth_2184 36% and to Moth_0978 25%. The fourth gene of the cluster, *ech1F*, encodes another subunit of Ech complexes. It has 24% identity to the corresponding subunit of *M. barkeri* and 26% to the homologous subunits (Moth_2185 and Moth_0982, respectively) from *M. thermoacetica*. It is predicted to have two 4Fe-4S clusters and therefore might be involved in electron transfer from ferredoxin. The following gene (*hycB1*/TKV_c01270) encodes a small FeS protein with four 4Fe-4S clusters that shows homology to HycB from *Escherichia coli* (33% identity) and CooF from *Rhodospirillum rubrum* (32% identity). Its identity to the homologous protein from *M. thermoacetica* (Moth_2192) is 36%. The protein products of the next two genes do not have any homologues in known Ech-type complexes, but show sequence similarity to the N-terminus of MetV, the small subunit of the methylene-THF reductase of the Wood-Ljungdahl pathway. The sequence identity to each other is 22%, the identity to MetV of *T. kivui* is 23% (for TKV_c01280) and 20% (for TKV_c01290), respectively. Both proteins might coordinate one 4Fe-4S cluster. 170 bp downstream of TKV_c01290 is a gene encoding the subunit Ech1D with 27% identity to the corresponding subunit from *M. barkeri*. It has no predicted cofactors and its function in the complex is unknown so far. The genome of *M. thermoacetica* does not code for a homologous protein. Adjacent lies the gene *ech1E*, encoding the catalytic NiFe hydrogenase subunit. The protein sequence shows 40% identity to EchE from *M. barkeri* and 39% and 33% to Moth_2186 and Moth_0980, respectively, from *M. thermoacetica*. A model of the protein complex Ech1 encoded by this *ech1* cluster is shown in Figure 5B. Electrons coming from reduced ferredoxin may enter the complex at subunit Ech1F and may be transferred *via* the FeS proteins HycB1, TKV_c01280 and TKV_c01290 onto the catalytic hydrogenase subunit Ech1E that catalyzes H_2_ formation. Subunits Ech1A and Ech1B mediate H^+^ transport across the cytoplasmic membrane.

The second gene cluster potentially encoding an Ech-type hydrogenase is TKV_c19750- TKV_c19680 (Figure 6A). The product of the first gene, *ech2D*, encodes a 14 kDa protein (Figure 6B) and is 19% identical to EchD from *M. barkeri*. The sequence identity to its homologue (TKV_c01300) in the Ech1 complex is only 8%. The succeeding gene, *ech2E*, codes for the large catalytic NiFe hydrogenase subunit and the deduced protein product shows 37% identity to *M. barkeri* EchE, 40% to Moth_2186 and 32% to Moth_0980 from *M. thermoacetica*, as well as 45% to TKV_c01310/Ech1E from *T. kivui*. Thereafter lies a gene encoding a 13 kDa protein (HycB2) with four predicted 4Fe-4S clusters. It has 35% identity to the FeS protein HycB1 (TKV_c01270) of the above described Ech-type complex and is homologous to CooF from *R. rubrum* (36% identity) and HycB from *E. coli* (39% identity), as well. The next two genes, TKV_c19720 and TKV_c19710, both encode proteins similar to the membrane-bound subunit EchA (with 22% and 21% identity to *M. barkeri* EchA, respectively and 18 to 27% identity to the six EchA homologues from *M. thermoacetica*) and were therefore named *ech2A1* and *ech2A2*. The homologue of Ech2A1 in complex I is NuoL, whereas the homologue of Ech2A2 is NuoM. Ech2C is encoded by the gene TKV_c19700. It has one hydrophobic stalk and represents the small hydrogenase subunit. Its identity to EchC from *M. barkeri* is 43%, *M. thermoacetica* Moth_2184 and Moth_0978 are 48% and 37% identical. The gene *ech2B* encodes another membrane-integral subunit with 7 transmembrane helices but no known cofactors. Its identity to Moth_0981 is 25%, sequence identity to Moth_2190 is only 17%. The last gene encodes another small electron transfer protein with two 4Fe-4S clusters and is similar to EchF, the identity to the corresponding proteins from *M. thermoacetica* is 28% (Moth_2185) and 24% (Moth_0982). A postulated subunit composition of the Ech2 complex and electron flow from reduced ferredoxin to H^+^ with concomitant H^+^ export is shown in Figure 6B.

Thus, in contrast to the above-described Ech-type complex, the second has one more membrane-integral subunit that might be involved in H^+^ transport, probably allowing for a more beneficial stoichiometry of the electron:H^+^ ratio. On the other hand, the small FeS proteins with similarity to MetV are missing. Sequence identities of the homologous proteins of the two Ech-type complexes to each other are summarized in Table 3.

**Table 3:** **Sequence identities of homologous subunits of the two Ech-type complexes from** ***T. kivui***

Locus tag 1	Locus tag 2	Protein subunit	Identity [%]
TKV_c19720	TKV_c01230	EchA	22
TKV_c19710	TKV_c01230	EchA	26
TKV_c19720	TKV_c19710	EchA	18
TKV_c19690	TKV_c01240	EchB	28
TKV_c19700	TKV_c01250	EchC	50
TKV_c19750	TKV_c01300	EchD	8
TKV_c19740	TKV_c01310	EchE	45
TKV_c19680	TKV_c01260	EchF	21
TKV_c19730	TKV_c01270	HycB	35

## Discussion

### Model of energy conservation during autotrophic growth of *T. kivui*

**Figure 7:**
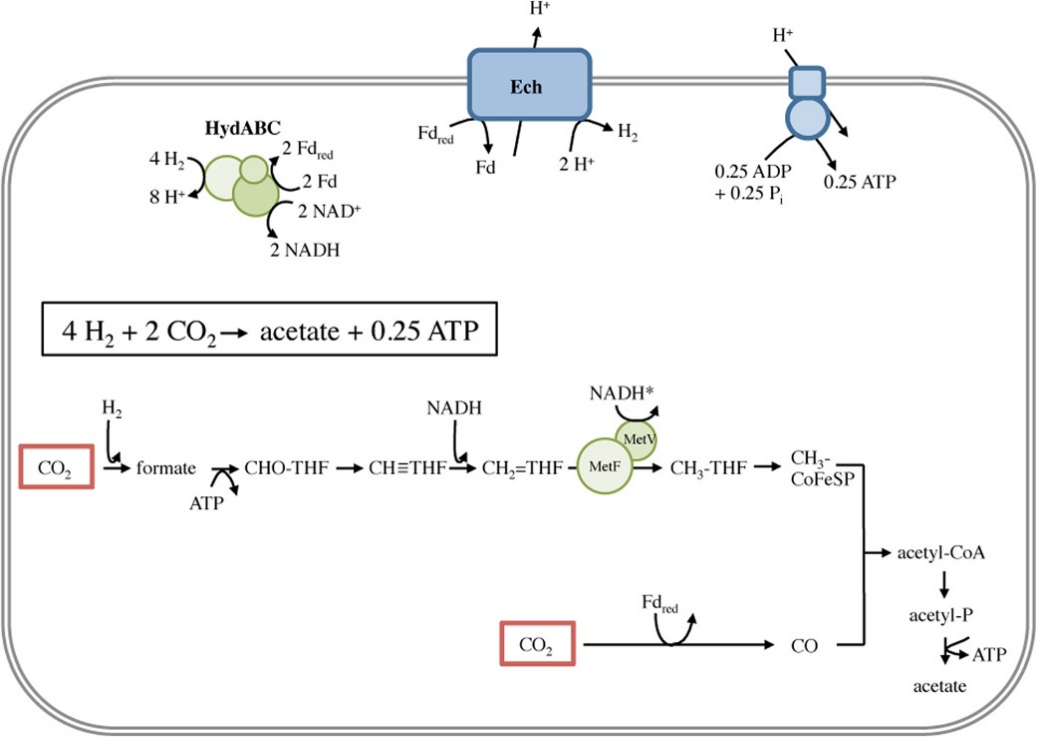
**Model of electron and carbon flow in** ***T. kivui*** **when growing autotrophically.** Fd_red_, reduced ferredoxin; a H^+^/ATP ratio of 12/3 was assumed for the F_1_F_O_ ATP synthase. *, reduction of methylene-THF might occur using an electron donor with a similar redox potential as NADH. CoFeSP, corrinoid/FeS protein.

The genomic data allow to propose a model for electron and carbon flow during acetogenesis from H_2_ + CO_2_ in *T. kivui*. Electrons coming from molecular hydrogen are transferred to the electron carriers NAD^+^ and ferredoxin by the soluble, electron-bifurcating hydrogenase HydABC. In the carbonyl branch of the WLP, the reduced ferredoxin serves as electron donor for the reduction of one molecule of CO_2_ to CO, catalyzed by the CODH/ACS. In the methyl branch of the pathway, another CO_2_ is reduced to formate with electrons coming directly from molecular hydrogen. This reaction is catalyzed by the hydrogen-dependent CO_2_ reductase (HDCR), as it was also shown for *A. woodii*. The electron donor for the subsequent reduction of methenyl-THF cannot be predicted, since both NADH and NADPH are used in other acetogens and bioinformatic analyses do not allow to discriminate between the possibilities. However, since NADH is more common, it was used as electron donor for the *T. kivui* model. The following step, reduction of methylene-THF is still under debate for an indirect role in energy conservation. The redox potential (E^0^’) of the methylene-THF/methyl-THF pair is −200 to −130 mV [72] and electron transfer from NADH (E^0^’ = −320 mV) to methylene-THF is highly exergonic. Therefore, this reaction was assumed already in 1977 to be involved in energy conservation [73]. One scenario is that methylene-THF reduction is coupled to ferredoxin reduction by electron bifurcation, as recently suggested for *M. thermoacetica* [31]. In *T. kivui*, MetV and MetF are encoded in the WLP operon (see Figure 1B), however, the genes encoding the putatively bifurcating Hdr subunits of *M. thermoacetica* are lacking in the *T. kivui* genome. Therefore, the situation is more similar to *A. woodii*, which is also lacking Hdr–encoding genes and in which evidence for electron bifurcation was not obtained (Bertsch J, Öppinger C, Hess V, Langer JD, Müller V: A heterotrimeric NADH-oxidizing methylenetetrahydrofolate reductase from the acetogenic bacterium Acetobacterium woodii, in preparation). Actually, MetV and MetF of *T. kivui* are the minimal subunit composition of an acetogenic, non-electron-bifurcating methylene-THF reductase. Since cell-free extract of *T. kivui* does not catalyze NADH- or NADPH-dependent methylene-THF reduction (data not shown) we assume an electron carrier in that redox range which may be, for example, a flavodoxin, which is energetically equivalent to NADH. If we take into account a non-electron-bifurcating methylene-THF reductase that uses the electrons derived from a carrier energetically equivalent to NADH we can conclude a quantitative scheme for the bioenergetics of acetogenesis in *T. kivui* (Figure 7). According to this model, *T. kivui* can synthesize 0.25 ATP *via* the F_1_F_O_ ATP synthase per acetate formed.

The ATP:acetate ratio in the phylogenetically close neighbor *M. thermoacetica* is predicted to be 0.5 [19], thus in comparison, *T. kivui* conserves only half as much energy from 4 H_2_ + 2 CO_2_. Both organisms have an electron bifurcating hydrogenase HydABC that couples initial H_2_ oxidation to the reduction of NAD^+^ and ferredoxin. The latter is the electron donor for the energy conserving reaction, the electron flow to H^+^*via* the Ech complex that concomitantly translocates protons across the cytoplasmic membrane. The above-mentioned difference in ATP production per acetate can be explained by the postulated additional coupling of methylene-THF reduction to the reduction of ferredoxin (or an energetic equivalent) in a second electron bifurcating reaction in *M. thermoacetica*. Hence this bacterium can transfer four electrons onto the Ech complex, thereby translocating two protons across the cytoplasmic membrane, whilst *T. kivui* uses the Ech complex only once per acetate formed.

As described in this paper, all data achieved are in accordance with H^+^ based bioenergetics in *T. kivui*. Based on bioinformatic and experimental data, we could show that the ATP synthase is H^+^-dependent and the electrochemical ion gradient is probably formed by a H^+^-translocating Ech type complex. However, these data are in contrast to the results obtained by Yang *et al.* in 1990 [35], who found a strict Na^+^ dependence when H_2_ was the electron donor. In order to assure our diverging results, we prepared our Na^+^-deficient medium strictly according to the instructions as given in [35]. We measured a contaminating Na^+^ concentration of 160 μM, while Yang and Drake reported 200 μM NaCl in their medium. Thus a different amount of Na^+^ in the deficient medium can be excluded as a source of the discrepancy. We reproduced this experiment several times and could never see an effect of Na^+^ on growth. Unfortunately, we were not able to find a reason for the discrepancy, but the additional data obtained in cell suspensions of *T. kivui* as well as the absence of a sodium ion binding site in the *c* subunit of the F_1_F_O_ ATP synthase are consistent with the classification of *T. kivui* as H^+^-dependent.

In the early history of acetogenic bacteria, *M. thermoacetica* was used as a model organism to unravel the mechanism of energy conservation in H^+^-dependent acetogens. The detection of cytochromes [74] led to the assumption that H^+^ translocation in *M. thermoacetica* is cytochrome-based [75–77]. However, despite many decades of intensive research, the participation of cytochromes in H^+^ translocation of *M. thermoacetica* could never be confirmed and their involvement in energy conservation became questionable. Instead, a role of cytochromes in O_2_ reduction was discussed in recent years [32]. With our finding that *T. kivui* and *M. thermoacetica* seem to use very similar mechanisms for energy conservation and that the genome of *T. kivui* does not encode any proteins necessary for cytochrome synthesis, it becomes more and more evident that the function of cytochromes in *M. thermoacetica* is indeed not in energy conservation coupled to the WLP. Instead, we have recently proposed to classify the acetogens bioenergetically in Rnf- and Ech-containing acetogens [19] and each class has a subclass with Na^+^- and H^+^-dependent species. *T. kivui* thus belongs to the subclass of H^+^-depending, Ech-containing acetogens.

By further comparing the two acetogens *T. kivui* and *M. thermoacetica*, it is remarkable that, despite similar mechanisms for energy conservation, their growth behavior on H_2_ + CO_2_ is quite different. As mentioned earlier, the doubling time of *M. thermoacetica* when growing autotrophically is about 24 h [32], while *T. kivui* grows 10-times faster [33]. But how can this discrepancy be explained in spite of the similar genetic configuration of the two bacteria? Since a weaker coupling of ATP synthesis and autotrophic acetate formation in *M. thermoacetica* was already excluded, the only explanation left is that at least one reaction of the Wood-Ljungdahl pathway is rate limiting. As *M. thermoacetica* is postulated to use three reactions that do not seem to be necessary in *T. kivui* (the transhydrogenase reaction for production of NADPH, reduction of CO_2_ with NADPH, and a bifurcating reaction in the course of methylene-THF reduction), one of those might well be the limiting factor. However, in order to irrevocably identify the bottleneck, extensive biochemical studies based on experimental data need to be carried out in future.

## Conclusions

Sequencing the genome of *T. kivui* allowed for a better insight into the reactions enabling the autotrophic reduction of CO_2_ to acetate. Since this process is thought to be one of the first pathways that evolved on the early earth [78], its detailed understanding is of great relevance.

The thermophilic acetogen *T. kivui* oxidizes the electron donor H_2_ by use of an electron bifurcating hydrogenase HydABC. The reduced ferredoxin then fuels the energy conserving membrane module that is coupled to the WLP: the genome of *T. kivui* harbors two gene clusters, which both encode Ech-type complexes. Although the deduced protein subunit composition of the two complexes alter slightly, both enzymes have all cofactors and catalytic domains that are required to catalyze ferredoxin oxidation as well as H^+^ reduction. The proton gradient formed by Ech can be used by a H^+^-dependent F_1_F_O_ ATP synthase to drive phosphorylation of ADP.

## Abbreviations

CODH/ACS: CO dehydrogenase/acetyl-CoA synthase
CoFeSP: Corrinoid/FeS protein
Ech: Energy conserving hydrogenase
ETH2120: Sodium ionophore III/N,N,N′,N′-Tetracyclohexyl-1,2-phenylenedioxydiacetamide
Fd: Ferredoxin
PTS: Phosphotransferase system
TCS: 4′,5-tetrachlorosalicylanilide
THF: Tetrahydrofolate
WLP: Wood-Ljungdahl pathway.

## References

[1] Drake HL, Gößner AS, Daniel SL (2008). Old acetogens, new light. Ann N Y Acad Sci.

[2] Müller V, Frerichs J, Battista J (2013). Acetogenic bacteria. eLS.

[3] Ljungdahl LG, Drake HL (1994). The acetyl-CoA pathway and the chemiosmotic generation of ATP during acetogenesis. Acetogenesis.

[4] Ragsdale SW (2008). Enzymology of the Wood-Ljungdahl pathway of acetogenesis. Ann N Y Acad Sci.

[5] Pezacka E, Wood HG (1984). Role of carbon monoxide dehydrogenase in the autotrophic pathway used by acetogenic bacteria. Proc Natl Acad Sci U S A.

[6] Raybuck SA, Bastian NR, Orme-Johnson WH, Walsh CT (1988). Kinetic characterization of the carbon monoxide-acetyl-CoA (carbonyl group) exchange activity of the acetyl-CoA synthesizing CO dehydrogenase from *Clostridium thermoaceticum*. Biochemistry.

[7] Seravalli J, Kumar M, Lu WP, Ragsdale SW (1997). Mechanism of carbon monoxide oxidation by the carbon monoxide dehydrogenase/acetyl-CoA synthase from *Clostridium thermoaceticum*: Kinetic characterization of the intermediates. Biochemistry.

[8] Himes RH, Harmony JA (1973). Formyltetrahydrofolate synthetase. Crc Cr Rev Bioch Mol.

[9] Lovell CR, Przybyla A, Ljungdahl LG (1988). Cloning and expression in *Escherichia coli* of the *Clostridium thermoaceticum* gene encoding thermostable formyltetrahydrofolate synthetase. Arch Microbiol.

[10] Ragsdale SW, Ljungdahl LG, DerVartanian DV (1982). EPR evidence for nickel-substrate interaction in carbon monoxide dehydrogenase from *Clostridium thermoaceticum*. Biochem Biophys Res Commun.

[11] Ragsdale SW, Ljungdahl LG, DerVartanian DV (1983). Isolation of carbon monoxide dehydrogenase from *Acetobacterium woodii* and comparison of its properties with those of the *Clostridium thermoaceticum* enzyme. J Bacteriol.

[12] Ragsdale SW, Wood HG (1985). Acetate biosynthesis by acetogenic bacteria. Evidence that carbon monoxide dehydrogenase is the condensing enzyme that catalyzes the final steps in the synthesis. J Biol Chem.

[13] Schaupp A, Ljungdahl LG (1974). Purification and properties of acetate kinase from *Clostridium thermoaceticum*. Arch Microbiol.

[14] Eden G, Fuchs G (1982). Total synthesis of acetyl coenzyme A involved in autotrophic CO_2_ fixation in *Acetobacterium woodii*. Arch Microbiol.

[15] Biegel E, Schmidt S, González JM, Müller V (2011). Biochemistry, evolution and physiological function of the Rnf complex, a novel ion-motive electron transport complex in prokaryotes. Cell Mol Life Sci.

[16] Biegel E, Müller V (2010). Bacterial Na^+^-translocating ferredoxin:NAD^+^ oxidoreductase. Proc Natl Acad Sci U S A.

[17] Heise R, Reidlinger J, Müller V, Gottschalk G (1991). A sodium-stimulated ATP synthase in the acetogenic bacterium *Acetobacterium woodii*. FEBS Lett.

[18] Müller V, Aufurth S, Rahlfs S (2001). The Na^+^ cycle in *Acetobacterium woodii*: identification and characterization of a Na^+^-translocating F_1_F_O_-ATPase with a mixed oligomer of 8 and 16 kDa proteolipids. Biochim Biophys Acta.

[19] Schuchmann K, Müller V (2014). Autotrophy at the thermodynamic limit of life: a model for energy conservation in acetogenic bacteria. Nat Rev Microbiol.

[20] Fröstl JM, Seifritz C, Drake HL (1996). Effect of nitrate on the autotrophic metabolism of the acetogens *Clostridium thermoautotrophicum* and *Clostridium thermoaceticum*. J Bacteriol.

[21] Seifritz C, Daniel SL, Gössner A, Drake HL (1993). Nitrate as a preferred electron sink for the acetogen *Clostridium thermoaceticum*. J Bacteriol.

[22] Dilling S, Imkamp F, Schmidt S, Müller V (2007). Regulation of caffeate respiration in the acetogenic bacterium *Acetobacterium woodii*. Appl Environ Microbiol.

[23] Misoph M, Daniel SL, Drake HL (1996). Bidirectional usage of ferulate by the acetogen *Peptostreptococcus productus* U-1: CO_2_ and aromatic acrylate groups as competing electron accepters. Microbiology-Uk.

[24] Gössner A, Daniel SL, Drake HL (1994). Acetogenesis coupled to the oxidation of aromatic aldehyde groups. Arch Microbiol.

[25] Matthies C, Freiberger A, Drake HL (1993). Fumarate dissimilation and differential reductant flow by *Clostridium formicoaceticum* and *Clostridium aceticum*. Arch Microbiol.

[26] Tanner RS, Miller LM, Yang D (1993). *Clostridium ljungdahlii* sp. nov., an acetogenic species in clostridial rRNA homology Group-I. Int J Syst Bact.

[27] Liou JS, Balkwill DL, Drake GR, Tanner RS (2005). *Clostridium carboxidivorans* sp. nov., a solvent-producing clostridium isolated from an agricultural settling lagoon, and reclassification of the acetogen *Clostridium scatologenes* strain SL1 as *Clostridium drakei* sp. nov. Int J Syst Evol Microbiol.

[28] Schiel-Bengelsdorf B, Dürre P (2012). Pathway engineering and synthetic biology using acetogens. FEBS Lett.

[29] Köpke M, Mihalcea C, Liew F, Tizard JH, Ali MS, Conolly JJ, Al-Sinawi B, Simpson SD (2011). 2,3-butanediol production by acetogenic bacteria, an alternative route to chemical synthesis, using industrial waste gas. Appl Environ Microbiol.

[30] Pierce E, Xie G, Barabote RD, Saunders E, Han CS, Detter JC, Richardson P, Brettin TS, Das A, Ljungdahl LG, Ragsdale SW (2008). The complete genome sequence of *Moorella thermoacetica* (f. *Clostridium thermoaceticum*). Environ Microbiol.

[31] Mock J, Wang S, Huang H, Kahnt J, Thauer RK (2014). Evidence for a hexaheteromeric methylenetetrahydrofolate reductase in *Moorella thermoacetica*. J Bacteriol.

[32] Huang H, Wang S, Moll J, Thauer RK (2012). Electron bifurcation involved in the energy metabolism of the acetogenic bacterium *Moorella thermoacetica* growing on glucose or H_2_ plus CO_2_. J Bacteriol.

[33] Leigh JA, Mayer F, Wolfe RS (1981). Acetogenium kivui, a new thermophilic hydrogen-oxidizing, acetogenic bacterium. Arch Microbiol.

[34] Daniel SL, Hsu T, Dean SI, Drake HL (1990). Characterization of the H_2_-dependent and CO-dependent chemolithotrophic potentials of the acetogens *Clostridium thermoaceticum* and *Acetogenium kivui*. J Bacteriol.

[35] Yang H, Drake HL (1990). Differential effects of sodium on hydrogen- and glucose-dependent growth of the acetogenic bacterium *Acetogenium kivui*. Appl Environ Microbiol.

[36] Bradford MM (1976). A rapid and sensitive method for the quantification of microgram quantities of protein utilizing the principle of proteine-dye-binding. Anal Biochem.

[37] Hess V, Schuchmann K, Müller V (2013). The ferredoxin:NAD^+^ oxidoreductase (Rnf) from the acetogen *Acetobacterium woodii* requires Na^+^ and is reversibly coupled to the membrane potential. J Biol Chem.

[38] Schönheit P, Wäscher C, Thauer RK (1978). A rapid procedure for the purification of ferredoxin from clostridia using polyethylenimine. FEBS Lett.

[39] Murray MG, Thompson WF (1980). Rapid isolation of high molecular weight plant DNA. Nucleic Acids Res.

[40] Wilson K, Ausubel FM (2001). Preparation of genomic DNA from bacteria. Current Protocols in Molecular Biology.

[41] Chevreux B (2005). MIRA: an automated genome and EST assembler.

[42] Darling AE, Mau B, Perna NT (2010). progressiveMauve: multiple genome alignment with gene gain, loss and rearrangement. PLoS One.

[43] Markowitz VM, Chen IM, Palaniappan K, Chu K, Szeto E, Pillay M, Ratner A, Huang J, Woyke T, Huntemann M, Anderson I, Billis K, Varghese N, Mavromatis K, Pati A, Ivanova NN, Kyrpides NC (2014). IMG 4 version of the integrated microbial genomes comparative analysis system. Nucleic Acids Res.

[44] Markowitz VM, Chen IM, Palaniappan K, Chu K, Szeto E, Grechkin Y, Ratner A, Jacob B, Huang J, Williams P, Huntemann M, Anderson I, Mavromatis K, Ivanova NN, Kyprides NC (2012). IMG: the Integrated Microbial Genomes database and comparative analysis system. Nucleic Acids Res.

[45] Tettelin H, Radune D, Kasif S, Khouri H, Salzberg SL (1999). Optimized multiplex PCR: efficiently closing a whole-genome shotgun sequencing project. Genomics.

[46] Staden R, Beal KF, Bonfield JK (2000). The Staden package, 1998. Methods Mol Biol.

[47] Hyatt D, Chen GL, Locascio PF, Land ML, Larimer FW, Hauser LJ (2010). Prodigal: prokaryotic gene recognition and translation initiation site identification. BMC Bioinformatics.

[48] Lagesen K, Hallin P, Rodland EA, Staerfeldt HH, Rognes T, Ussery DW (2007). RNAmmer: consistent and rapid annotation of ribosomal RNA genes. Nucleic Acids Res.

[49] Lowe TM, Eddy SR (1997). tRNAscan-SE: a program for improved detection of transfer RNA genes in genomic sequence. Nucleic Acids Res.

[50] Zdobnov EM, Apweiler R (2001). InterProScan–an integration platform for the signature-recognition methods in InterPro. Bioinformatics.

[51] Zhou Y, Liang Y, Lynch KH, Dennis JJ, Wishart DS (2011). PHAST: a fast phage search tool. Nucleic Acids Res.

[52] Schmidt K, Liaaen Jensen S, Schlegel HG (1963). Die Carotinoide der *Thiorhodaceae.* I Okenon als Hauptcarotinoid von *Chromatium okenii* Perty. Arch Mikrobiol.

[53] Horvath P, Barrangou R (2010). CRISPR/Cas, the immune system of bacteria and archaea. Science.

[54] Haft DH, Selengut J, Mongodin EF, Nelson KE (2005). A guild of 45 CRISPR-associated (Cas) protein families and multiple CRISPR/Cas subtypes exist in prokaryotic genomes. PLoS Comput Biol.

[55] Westra ER, Brouns SJ (2012). The rise and fall of CRISPRs–dynamics of spacer acquisition and loss. Mol Microbiol.

[56] Makarova KS, Haft DH, Barrangou R, Brouns SJ, Charpentier E, Horvath P, Moineau S, Mojica FJ, Wolf YI, Yakunin AF, van der Oost J, Koonin EV (2011). Evolution and classification of the CRISPR-Cas systems. Nat Rev Microbiol.

[57] Poehlein A, Schmidt S, Kaster A-K, Goenrich M, Vollmers J, Thürmer A, Bertsch J, Schuchmann K, Voigt B, Hecker M, Daniel R, Thauer RK, Gottschalk G, Müller V (2012). An ancient pathway combining carbon dioxide fixation with the generation and utilization of a sodium ion gradient for ATP synthesis. PLoS One.

[58] Schuchmann K, Müller V (2013). Direct and reversible hydrogenation of CO_2_ to formate by a bacterial carbon dioxide reductase. Science.

[59] Li LF, Ljungdahl L, Wood HG (1966). Properties of nicotinamide adenine dinucleotide phosphate-dependent formate dehydrogenase from *Clostridium thermoaceticum*. J Bacteriol.

[60] Schuchmann K, Müller V (2012). A bacterial electron bifurcating hydrogenase. J Biol Chem.

[61] Schut GJ, Adams MW (2009). The iron-hydrogenase of *Thermotoga maritima* utilizes ferredoxin and NADH synergistically: a new perspective on anaerobic hydrogen production. J Bacteriol.

[62] Wang S, Huang H, Kahnt J, Thauer RK (2013). A reversible electron-bifurcating ferredoxin- and NAD-dependent [FeFe]-hydrogenase (HydABC) in *Moorella thermoacetica*. J Bacteriol.

[63] Müller V, Grüber G (2003). ATP synthases: structure, function and evolution of unique energy converters. Cell Mol Life Sci.

[64] Brandt K, Müller DB, Hoffmann J, Hübert C, Brutschy B, Deckers-Hebestreit G, Müller V (2013). Functional production of the Na^+^ F_1_F_O_ ATP synthase from *Acetobacterium woodii* in *Escherichia coli* requires the native AtpI. J Bioenerg Biomembr.

[65] Rahlfs S, Aufurth S, Müller V (1999). The Na^+^-F_1_F_O_-ATPase operon from *Acetobacterium woodii*. Operon structure and presence of multiple copies of *atpE* which encode proteolipids of 8- and 18-kDa. J Biol Chem.

[66] Meier T, Krah A, Bond PJ, Pogoryelov D, Diederichs K, Faraldo-Gómez JD (2009). Complete ion-coordination structure in the rotor ring of Na^+^-dependent F-ATP synthases. J Mol Biol.

[67] Hedderich R (2004). Energy-converting [NiFe] hydrogenases from archaea and extremophiles: ancestors of complex I. J Bioenerg Biomembr.

[68] Welte C, Krätzer C, Deppenmeier U (2010). Involvement of Ech hydrogenase in energy conservation of *Methanosarcina mazei*. FEBS J.

[69] Meuer J, Bartoschek S, Koch J, Künkel A, Hedderich R (1999). Purification and catalytic properties of Ech hydrogenase from *Methanosarcina barkeri*. Eur J Biochem.

[70] Welte C, Deppenmeier U (2013). Bioenergetics and anaerobic respiratory chains of aceticlastic methanogens. Biochim Biophys Acta.

[71] Hedderich R, Forzi L (2005). Energy-converting [NiFe] hydrogenases: more than just H_2_ activation. J Mol Microbiol Biotechnol.

[72] Maden BE (2000). Tetrahydrofolate and tetrahydromethanopterin compared: functionally distinct carriers in C_1_ metabolism. Biochem J.

[73] Thauer RK, Jungermann K, Decker K (1977). Energy conservation in chemotrophic anaerobic bacteria. Bact Rev.

[74] Gottwald M, Andreesen JR, LeGall J, Ljungdahl LG (1975). Presence of cytochrome and menaquinone in *Clostridium formicoaceticum* and *Clostridium thermoaceticum*. J Bacteriol.

[75] Hugenholtz J, Ivey DM, Ljungdahl LG (1987). Carbon monoxide-driven electron transport in *Clostridium thermoautotrophicum* membranes. J Bacteriol.

[76] Hugenholtz J, Ljungdahl LG (1989). Electron transport and electrochemical proton gradient in membrane vesicles of *Clostridium thermoaceticum*. J Bacteriol.

[77] Hugenholtz J, Ljungdahl LG (1990). Amino acid transport in membrane vesicles of *Clostridium thermoautotrophicum*. FEMS Microbiol Lett.

[78] Martin WF (2012). Hydrogen, metals, bifurcating electrons, and proton gradients: the early evolution of biological energy conservation. FEBS Lett.

